# Bis[μ-2-(pyridin-2-yl)ethano­lato]bis­[bromidocopper(II)]

**DOI:** 10.1107/S1600536811043637

**Published:** 2011-10-29

**Authors:** M. Mobin Shaikh, Saloni Mathur, Md. Jamal Uddin

**Affiliations:** a National Single Crystal X-ray Diffraction Facility, IIT Bombay, Powai, Mumbai 400 076, India; b Department of Natural Sciences, Coppin State University, 2500 West North Avenue, Baltimore, Maryland 21216, USA

## Abstract

The title compound, [Cu_2_Br_2_(C_7_H_8_NO)_2_], was synthesized by reaction of CuBr_2_ with 2-(pyridin-2-yl)ethanol (hep-H) in methanol. The asymmetric unit consists of one hep ligand and a CuBr unit. The Cu^2+^ ion is thereby coordinated by the N atom and the deprotonated hydroxy O atom in a distorted square-planar geometry that is completed by another O atom. The latter acts as bridging ligand towards the second, symmetry-equivalent, Cu atom, thus generating a centrosymmetric dimeric unit, with the inversion centre halfway between the Cu atoms. These units are linked *via* C—H⋯Br and C—H⋯O hydrogen bonds, leading to the formation of a hydrogen-bonded one-dimensional-polymeric chain along *a*..

## Related literature

For similar dinuclear copper complexes see Lah *et al.* (2006 ▶); Shaikh *et al.* (2010 ▶).
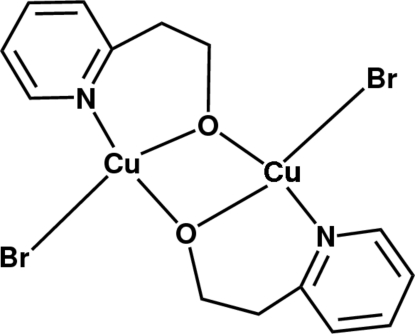


## Experimental

### 

#### Crystal data

[Cu_2_Br_2_(C_7_H_8_NO)_2_]
*M* *_r_* = 531.19
Triclinic, 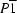

*a* = 4.2066 (2) Å
*b* = 8.4338 (3) Å
*c* = 11.5113 (6) Å
α = 91.122 (4)°
β = 90.195 (3)°
γ = 97.033 (1)°
*V* = 405.24 (3) Å^3^
*Z* = 1
Mo *K*α radiation
μ = 7.56 mm^−1^
*T* = 150 K
0.28 × 0.21 × 0.17 mm

#### Data collection

Oxford Diffraction Xcalibur-S diffractometer
Absorption correction: multi-scan (*CrysAlis RED*; Oxford Diffraction, 2009 ▶)*T*_min_ = 0.226, *T*_max_ = 0.360
3453 measured reflections
1388 independent reflections
1298 reflections with *I* > 2σ(*I*)
*R*_int_ = 0.026

#### Refinement

*R*[*F* ^2^ > 2σ(*F* ^2^)] = 0.031
*wR*(*F* ^2^) = 0.087
*S* = 1.05
1388 reflections
100 parameters
H-atom parameters constrained
Δρ_max_ = 0.84 e Å^−3^
Δρ_min_ = −0.74 e Å^−3^

### 

Data collection: *CrysAlis CCD* (Oxford Diffraction, 2009 ▶); cell refinement: *CrysAlis CCD*; data reduction: *CrysAlis RED* (Oxford Diffraction, 2009 ▶); program(s) used to solve structure: *SHELXS97* (Sheldrick, 2008 ▶); program(s) used to refine structure: *SHELXL97* (Sheldrick, 2008 ▶); molecular graphics: *DIAMOND* (Brandenburg, 1999 ▶); software used to prepare material for publication: *publCIF* (Westrip, 2010 ▶).

## Supplementary Material

Crystal structure: contains datablock(s) I, global. DOI: 10.1107/S1600536811043637/fi2115sup1.cif

Structure factors: contains datablock(s) I. DOI: 10.1107/S1600536811043637/fi2115Isup2.hkl

Additional supplementary materials:  crystallographic information; 3D view; checkCIF report

## Figures and Tables

**Table:** 

Cu1—O1^i^	1.910 (3)
Cu1—O1	1.943 (3)
Cu1—N1	1.977 (3)
Cu1—Br1	2.3670 (6)
Cu1—Cu1^i^	3.0294 (9)

**Table:** 

O1^i^—Cu1—O1	76.32 (12)
Cu1^i^—O1—Cu1	103.68 (12)

Symmetry code: (i) 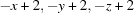.

**Table 2:** Hydrogen-bond geometry (Å, °)

*D*—H⋯*A*	*D*—H	H⋯*A*	*D*⋯*A*	*D*—H⋯*A*
C1—H1⋯Br1^ii^	0.95	3.00	3.716 (4)	134
C6—H6*A*⋯O1^iii^	0.99	2.64	3.545 (5)	153

Symmetry codes: (ii) 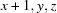; (iii) 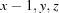.

**Fig. 1.**
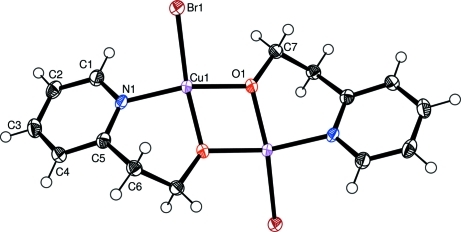
View of the molecular structure of the title compound with displacement ellipsoids drawn at the 50% probability level. Symmetry-related moiety generated by i: –x, –y, –z.

**Fig. 2.**
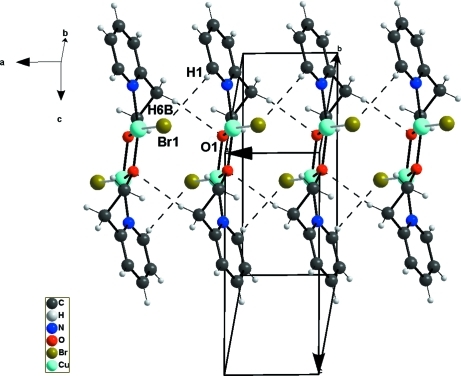
A tilted perspective view of hydrogen bonded one-dimensional-polymeric chain along a-axis. Hydrogen bonds as dashed lines.

## supplementary crystallographic information

### Comment

Dinuclear Cu(II) complexes have often been used as models to study the magnetic-exchange interactions and as building blocks for the construction of polynuclear compounds with interesting magnetic properties (Lah *et al.* 2006). The alkoxo bridged dinuclear Cu(II) complexes consists of two copper atoms bridged by two alkoxido oxygen atoms from alkoxypyridine-type ligands have drawn considerable interest in solid state transformations (Shaikh *et al.* 2010).

The dimeric title compound (Fig.1) features a dinuclear complex with site symmetry –1. The Cu (II) ions are linked via the two µ^2^-alcoholic oxygen atoms, yielding a four-membered planar ring Cu_2_O_2_. One pyridine nitrogen atom of hep and the bromide ligands complete the coordination environment, yielding a distorted square-planar geometry. The Cu ions are separated by 3.0294 (9) Å. The µ-O bridge is slightly asymmetric with Cu—O distances of 1.910 (3) and 1.943 (3) Å and Cu—O—Cu angle of 103.68°. (Table 1). These bond-distances and angles are in agreement with the reported dimeric molecules by Lah *et al.* (2006) and Shaikh *et al.* (2010).

Moreover, each dimeric unit is further extended through C—H···Br and C—H···O hydrogen bondings (Table 2) with the neighboring dimeric unit forming a one-dimensional-polymeric chains along *a*-axis (Fig. 2).

### Experimental

A solution of hep-H (123 mg, 1.0 mmol) in 30 ml methanol was added to a 10 ml methanolic solution of CuBr_2_ (223 mg, 1.0 mmol) and the resultant solution was stirred for 2 h at room temperature. The solution was then passed through filter paper (Whatman filter paper, 70 mm) in order to remove any unreacted materials. The filtrate was allowed to stand at room temperature for crystallization. On slow evaporation light blue single crystals of [Cu(µ-hep)Br]_2_ were obtained after 10 days. M.P.:488–490 K. Yield: 82%. Anal. Calcd for C_14_H_16_Br_2_Cu_2_N_2_O_2_ (Mr = 531.19): C,31.66; H, 3.04; N, 5.27. Found: C,31.30; H,3.11; N, 5.67.

### Refinement

The hydrogen atoms were placed geometrically and treated as riding on their parent atoms, with C—H 0.95 (pyridyl), C—H 0.99 (methylene) Å [*U*_iso_(H) = 1.2*U*_eq_(C)].

### Crystal data

**Table:**

[Cu_2_Br_2_(C_7_H_8_NO)_2_]	*Z* = 1
*M**_r_* = 531.19	*F*(000) = 258
Triclinic, *P*1	*D*_x_ = 2.177 Mg m^−^^3^
Hall symbol: -P 1	Mo *K*α radiation, λ = 0.71073 Å
*a* = 4.2066 (2) Å	Cell parameters from 3586 reflections
*b* = 8.4338 (3) Å	θ = 3.5–30.0°
*c* = 11.5113 (6) Å	µ = 7.56 mm^−^^1^
α = 91.122 (4)°	*T* = 150 K
β = 90.195 (3)°	Block, blue
γ = 97.033 (1)°	0.28 × 0.21 × 0.17 mm
*V* = 405.24 (3) Å^3^	

### Data collection

**Table:**

Oxford Diffraction Xcalibur-S diffractometer	1388 independent reflections
Radiation source: Enhance (Mo) X-ray Source	1298 reflections with *I* > 2σ(*I*)
graphite	*R*_int_ = 0.026
Detector resolution: 15.9948 pixels mm^-1^	θ_max_ = 25.0°, θ_min_ = 3.5°
ω/q scans	*h* = −5→4
Absorption correction: multi-scan (*CrysAlis PRO*-RED; Oxford Diffraction, 2009)	*k* = −9→9
*T*_min_ = 0.226, *T*_max_ = 0.360	*l* = −13→13
3453 measured reflections	

### Refinement

**Table:**

Refinement on *F*^2^	Primary atom site location: structure-invariant direct methods
Least-squares matrix: full	Secondary atom site location: difference Fourier map
*R*[*F*^2^ > 2σ(*F*^2^)] = 0.031	Hydrogen site location: inferred from neighbouring sites
*wR*(*F*^2^) = 0.087	H-atom parameters constrained
*S* = 1.05	*w* = 1/[σ^2^(*F*_o_^2^) + (0.0549*P*)^2^ + 0.5089*P*] where *P* = (*F*_o_^2^ + 2*F*_c_^2^)/3
1388 reflections	(Δ/σ)_max_ = 0.001
100 parameters	Δρ_max_ = 0.84 e Å^−^^3^
0 restraints	Δρ_min_ = −0.74 e Å^−^^3^

### Special details

**Table:**

Geometry. All esds (except the esd in the dihedral angle between two l.s. planes) are estimated using the full covariance matrix. The cell esds are taken into account individually in the estimation of esds in distances, angles and torsion angles; correlations between esds in cell parameters are only used when they are defined by crystal symmetry. An approximate (isotropic) treatment of cell esds is used for estimating esds involving l.s. planes.
Refinement. Refinement of F^2^ against ALL reflections. The weighted R-factor wR and goodness of fit S are based on F^2^, conventional R-factors R are based on F, with F set to zero for negative F^2^. The threshold expression of F^2^ > 2sigma(F^2^) is used only for calculating R-factors(gt) etc. and is not relevant to the choice of reflections for refinement. R-factors based on F^2^ are statistically about twice as large as those based on F, and R- factors based on ALL data will be even larger.

### Fractional atomic coordinates and isotropic or equivalent isotropic displacement parameters (Å^2^)

**Table:**

	*x*	*y*	*z*	*U*_iso_*/*U*_eq_	
Cu1	0.91306 (11)	0.96968 (5)	0.87303 (4)	0.01736 (18)	
Br1	0.60658 (9)	0.76112 (4)	0.76674 (3)	0.02191 (18)	
O1	1.0535 (7)	1.1399 (3)	0.9846 (2)	0.0219 (6)	
N1	1.0035 (8)	1.1189 (4)	0.7436 (3)	0.0174 (7)	
C1	1.1498 (10)	1.0720 (5)	0.6475 (4)	0.0220 (9)	
H1	1.1873	0.9635	0.6396	0.026*	
C2	1.2475 (10)	1.1759 (5)	0.5598 (4)	0.0254 (9)	
H2	1.3495	1.1398	0.4925	0.030*	
C3	1.1933 (10)	1.3341 (5)	0.5722 (4)	0.0266 (10)	
H3	1.2575	1.4082	0.5131	0.032*	
C4	1.0444 (10)	1.3833 (5)	0.6716 (4)	0.0226 (9)	
H4	1.0082	1.4917	0.6817	0.027*	
C5	0.9486 (9)	1.2720 (5)	0.7564 (3)	0.0186 (8)	
C6	0.7862 (9)	1.3159 (5)	0.8657 (4)	0.0197 (8)	
H6A	0.5811	1.2456	0.8729	0.024*	
H6B	0.7365	1.4273	0.8605	0.024*	
C7	0.9894 (10)	1.3012 (4)	0.9745 (3)	0.0195 (8)	
H7A	1.1934	1.3728	0.9691	0.023*	
H7B	0.8737	1.3335	1.0441	0.023*	

### Atomic displacement parameters (Å^2^)

**Table:**

	*U*^11^	*U*^22^	*U*^33^	*U*^12^	*U*^13^	*U*^23^
Cu1	0.0243 (3)	0.0158 (3)	0.0116 (3)	0.0008 (2)	0.0009 (2)	0.0016 (2)
Br1	0.0253 (3)	0.0211 (3)	0.0183 (3)	−0.00140 (17)	−0.00193 (18)	0.00043 (17)
O1	0.0347 (17)	0.0151 (14)	0.0161 (15)	0.0033 (12)	−0.0002 (12)	0.0032 (11)
N1	0.0201 (17)	0.0206 (17)	0.0117 (17)	0.0031 (13)	−0.0019 (13)	0.0001 (13)
C1	0.025 (2)	0.024 (2)	0.018 (2)	0.0060 (16)	0.0004 (17)	0.0012 (17)
C2	0.025 (2)	0.035 (2)	0.016 (2)	0.0017 (18)	0.0049 (17)	−0.0003 (18)
C3	0.028 (2)	0.029 (2)	0.022 (2)	−0.0008 (18)	0.0017 (18)	0.0086 (18)
C4	0.027 (2)	0.019 (2)	0.021 (2)	−0.0008 (16)	−0.0011 (17)	0.0030 (16)
C5	0.0153 (19)	0.023 (2)	0.018 (2)	0.0019 (15)	−0.0031 (15)	0.0012 (16)
C6	0.020 (2)	0.0186 (19)	0.021 (2)	0.0043 (16)	0.0010 (16)	0.0007 (16)
C7	0.023 (2)	0.0172 (19)	0.018 (2)	0.0034 (15)	0.0048 (16)	0.0000 (16)

### Geometric parameters (Å, °)

**Table:**

Cu1—O1^i^	1.910 (3)	C2—H2	0.9500
Cu1—O1	1.943 (3)	C3—C4	1.387 (6)
Cu1—N1	1.977 (3)	C3—H3	0.9500
Cu1—Br1	2.3670 (6)	C4—C5	1.394 (6)
Cu1—Cu1^i^	3.0294 (9)	C4—H4	0.9500
O1—C7	1.426 (4)	C5—C6	1.496 (6)
O1—Cu1^i^	1.910 (3)	C6—C7	1.529 (6)
N1—C5	1.344 (5)	C6—H6A	0.9900
N1—C1	1.344 (5)	C6—H6B	0.9900
C1—C2	1.380 (6)	C7—H7A	0.9900
C1—H1	0.9500	C7—H7B	0.9900
C2—C3	1.385 (6)		
			
O1^i^—Cu1—O1	76.32 (12)	C2—C3—C4	119.4 (4)
O1^i^—Cu1—N1	162.34 (14)	C2—C3—H3	120.3
O1—Cu1—N1	90.44 (12)	C4—C3—H3	120.3
O1^i^—Cu1—Br1	98.08 (8)	C3—C4—C5	119.3 (4)
O1—Cu1—Br1	163.87 (9)	C3—C4—H4	120.4
N1—Cu1—Br1	97.69 (10)	C5—C4—H4	120.4
O1^i^—Cu1—Cu1^i^	38.54 (8)	N1—C5—C4	120.8 (4)
O1—Cu1—Cu1^i^	37.78 (8)	N1—C5—C6	116.9 (3)
N1—Cu1—Cu1^i^	127.28 (10)	C4—C5—C6	122.4 (4)
Br1—Cu1—Cu1^i^	134.80 (3)	C5—C6—C7	112.9 (3)
C7—O1—Cu1^i^	125.6 (2)	C5—C6—H6A	109.0
C7—O1—Cu1	124.4 (2)	C7—C6—H6A	109.0
Cu1^i^—O1—Cu1	103.68 (12)	C5—C6—H6B	109.0
C5—N1—C1	119.7 (3)	C7—C6—H6B	109.0
C5—N1—Cu1	119.9 (3)	H6A—C6—H6B	107.8
C1—N1—Cu1	120.0 (3)	O1—C7—C6	109.4 (3)
N1—C1—C2	122.3 (4)	O1—C7—H7A	109.8
N1—C1—H1	118.9	C6—C7—H7A	109.8
C2—C1—H1	118.9	O1—C7—H7B	109.8
C1—C2—C3	118.5 (4)	C6—C7—H7B	109.8
C1—C2—H2	120.8	H7A—C7—H7B	108.2
C3—C2—H2	120.8		
			
O1^i^—Cu1—O1—C7	−153.1 (4)	Cu1—N1—C1—C2	−173.3 (3)
N1—Cu1—O1—C7	38.7 (3)	N1—C1—C2—C3	0.3 (6)
Br1—Cu1—O1—C7	−81.8 (4)	C1—C2—C3—C4	0.2 (6)
Cu1^i^—Cu1—O1—C7	−153.1 (4)	C2—C3—C4—C5	−0.8 (6)
O1^i^—Cu1—O1—Cu1^i^	0.0	C1—N1—C5—C4	−0.6 (6)
N1—Cu1—O1—Cu1^i^	−168.20 (15)	Cu1—N1—C5—C4	172.6 (3)
Br1—Cu1—O1—Cu1^i^	71.3 (3)	C1—N1—C5—C6	−179.7 (4)
O1^i^—Cu1—N1—C5	−77.1 (5)	Cu1—N1—C5—C6	−6.5 (5)
O1—Cu1—N1—C5	−36.2 (3)	C3—C4—C5—N1	1.1 (6)
Br1—Cu1—N1—C5	129.8 (3)	C3—C4—C5—C6	−179.9 (4)
Cu1^i^—Cu1—N1—C5	−45.2 (3)	N1—C5—C6—C7	65.4 (5)
O1^i^—Cu1—N1—C1	96.1 (5)	C4—C5—C6—C7	−113.7 (4)
O1—Cu1—N1—C1	137.0 (3)	Cu1^i^—O1—C7—C6	−145.3 (3)
Br1—Cu1—N1—C1	−56.9 (3)	Cu1—O1—C7—C6	1.9 (4)
Cu1^i^—Cu1—N1—C1	128.0 (3)	C5—C6—C7—O1	−60.6 (4)
C5—N1—C1—C2	−0.1 (6)		

Symmetry codes: (i) −*x*+2, −*y*+2, −*z*+2.

### Hydrogen-bond geometry (Å, °)

**Table:**

*D*—H···*A*	*D*—H	H···*A*	*D*···*A*	*D*—H···*A*
C1—H1···Br1^ii^	0.95	3.00	3.716 (4)	134.
C6—H6A···O1^iii^	0.99	2.64	3.545 (5)	153.

Symmetry codes: (ii) *x*+1, *y*, *z*; (iii) *x*−1, *y*, *z*.
